# Myeloid-Derived Suppressive Cells Deficient in Liver X Receptor α Protected From Autoimmune Hepatitis

**DOI:** 10.3389/fimmu.2021.732102

**Published:** 2021-08-26

**Authors:** Bo Li, Min Lian, Yikang Li, Qiwei Qian, Jun Zhang, Qiaoyan Liu, Ruqi Tang, Xiong Ma

**Affiliations:** Division of Gastroenterology and Hepatology, Key Laboratory of Gastroenterology and Hepatology, Ministry of Health, State Key Laboratory for Oncogenes and Related Genes, Renji Hospital, School of Medicine, Shanghai JiaoTong University, Shanghai Institute of Digestive Disease, Shanghai, China

**Keywords:** liver X receptor α, myeloid-derived suppressor cells, autoimmune hepatitis, interferon regulatory factor 8, immune-mediated hepatitis

## Abstract

Myeloid-derived suppressor cells (MDSCs) emerge as a promising candidate for the immunotherapy of autoimmune hepatitis (AIH). However, targets for modulating MDSC in AIH are still being searched. Liver X receptors (LXRs) are important nuclear receptors linking lipid metabolism and immune responses. Despite the extensive studies of LXR in myeloid compartment, its role in MDSCs is currently less understood. Herein, expression of LXRα was found to be upregulated in AIH patients and colocalized with hepatic MDSCs. In ConA-induced hepatitis, deletion of LXRα led to increased expansion of MDSCs in the liver and alleviated the hepatic injury. MDSCs in LXRα^−/−^ mice exhibited enhanced proliferation and survival comparing with WT mice. T-cell proliferation assay and adoptive cell transfer experiment validated the potent immunoregulatory role of MDSCs *in vitro* and *in vivo*. Mechanistically, MDSCs from LXRα^−/−^ mice possessed significantly lower expression of interferon regulatory factor 8 (IRF-8), a key negative regulator of MDSC differentiation. Transcriptional activation of IRF-8 by LXRα was further demonstrated

**Conclusion:**

We reported that abrogation of LXRα facilitated the expansion of MDSCs *via* downregulating IRF-8, and thereby ameliorated hepatic immune injury profoundly. Our work highlights the therapeutic potential of targeting LXRα in AIH.

## Introduction

Autoimmune hepatitis (AIH) is an autoimmune liver disease characterized by immune-mediated destruction of hepatocytes and accumulation of autoantibodies. Massive infiltration of CD4^+^ T lymphocytes in the liver of AIH and a genetic predisposition linked to HLA class II suggested a predominant role of CD4^+^ T cells in AIH (1). In murine model, administration of concanavalin (ConA) leads to apoptotic and necrotic liver injury, accompanied by marked elevation of interferon-γ (IFN-γ) and tumor necrosis factor-α (TNF-α), which resemble the immunopathology of AIH (2).

Myeloid-derived suppressor cells (MDSCs) are a heterogeneous population of immature myeloid cells that are known for their potent immunosuppressive abilities (3). In mice, MDSCs (CD11b^+^Gr1^+^) are categorized into two major subsets based on the cell morphology and phenotype and are commonly defined as polymorphonuclear (PMN)-MDSC (CD11b^+^Ly6G^+^Ly6C^lo^) and monocytic (M)-MDSC (CD11b^+^Ly6G^−^Ly6C^high^) (3). MDSCs have been demonstrated to protect liver from immune injury in inflammatory murine models, by inhibiting proliferation and cytotoxicity of T lymphocytes (4–7). Moreover, evidence provided by us and others described the clinical significance of MDSCs in autoimmune liver diseases (AILD), including AIH and primary biliary cholangitis (PBC), indicating MDSCs as a potential target for the immunotherapy of AILD (8–10).

Liver X receptors (LXRs) are members of nuclear receptors (NRs) activated by derivatives of cholesterol and emerge as an essential link between lipid metabolism and immune responses (10, 11). Interestingly, LXR has been implicated to orchestrate the fate of myeloid cells (12). It has been established that activation of LXRα substantially blunted the inflammatory responses of macrophage to LPS stimulation, mainly *via* inhibiting gene transcription by NF-κB and AP-1 (12, 13). However, it was subsequently reported that long-term exposure to LXR agonist in turn potentiated the LPS response (14), and in accordance, treatment of LXR agonist increased IL-1β expression in human macrophage by transactivating HIF-1α (15). In addition, activation of LXR sensitized human dendritic cells (DCs) to inflammatory stimulation (16), while endogenous LXR ligands produced within tumor sites were found to dampen DC migration and favored immunosuppressive function (17). With regard to neutrophils, activation of LXRs impaired the chemotactic and killing capacities of neutrophils during sepsis (18).

Although LXR has been extensively studied in myeloid compartment, its role in MDSCs is largely unknown. Herein, we present data showing that deletion of LXRα favored the differentiation and survival of MDSCs by downregulating interferon regulatory factor 8 (IRF-8), and consequently prevented ConA-induced liver injury. Furthermore, LXRα is highly expressed in AIH patients, which highlights the therapeutic value of LXRα suppression in AIH.

## Materials and Methods

### Patients

AIH patients were diagnosed according to the criteria established by the International Autoimmune Hepatitis Group in 2008 (19). The clinical characteristics of AIH patients and healthy controls who provided peripheral blood samples are listed in **Supplementary Table S1**.

All the AIH patients enrolled provided written informed consent, and the study was approved by the Ethics Committee of Renji Hospital.

### Liver Histology and Immunostaining

Immunohistochemistry and immunofluorescence were performed using primary antibodies against LXRα (ab41902, Abcam, Cambridge, UK), CD11b (ab238794, Abcam), and CD33 (ab199432, Abcam), according to the procedures described previously (8). Specifically, liver frozen sections of AIH patients who received liver transplantation were used for immunostaining of LXRα. Redundant liver explants from healthy donors were used as controls.

For murine experiment, liver tissue samples were fixed in 10% neutral buffered formalin and embedded in paraffin wax. Liver sections (4 μm) were stained with hematoxylin and eosin (H&E) for histological evaluation.

### Single-Cell RNA Sequencing Data

Public single-cell RNA sequencing data of hepatic nonparenchymal cells from healthy controls (*n* = 5) and patients of liver cirrhosis (*n* = 5) were downloaded from GEO dataset (GSE136103) (20). Hepatic expression of NR1H3 (encoding LXRα) was analyzed using *Seurat* R package v3.2.1 (21). Different immune lineages were identified with CellMarker dataset (22).

### Mice

Wild-type (WT) C57BL/6J were purchased from the Shanghai SLAC Laboratory Animal Co. Ltd. LXRα^−/−^ mice on a C57BL/6 background were kindly provided by professor Jun Pu (Division of Cardiology, Renji Hospital). All the mice were housed under specific pathogen-free (SPF) environment at the animal facility of Renji Hospital, School of Medicine, Shanghai Jiao Tong University. Female mice aged between 8 and 10 weeks were used. All animal experiments were reviewed and approved by the Institutional Animal Care and Use Committee of Shanghai Jiao Tong University.

### Acute Hepatitis Model

To induce acute hepatitis, LXRα^−/−^ mice and WT controls were i.v. injected with PBS or 8–10 mg/kg ConA (Sigma-Aldrich, St. Louis, MO, USA), respectively. In an attempt to antagonize the activation of LXR, WT mice were given SR9243 (30 mg/kg, SelleckChem, Houston, TX, USA) intraperitoneally twice at 24 and 1 h before ConA treatment. Mice were killed 24 h following ConA challenge to examine tissue injury, serum alanine aminotransferase (ALT), and aspartate aminotransferase (AST).

### Measurement of Serum Cytokines

Serum levels of interferon-γ (IFN-γ), tumor necrosis factor-α (TNF-α), and interleukin-6 (IL-6) were measured with Mouse Th1/Th2/Th17 Cytokine Kit (BD Bioscience, San Jose, CA, USA).

### Cell Preparation

Hepatic mononuclear cells (HMNCs) were prepared as previously described (7). Briefly, the liver was diced and homogenized by passed through a 70-μm strainer (BD Bioscience, USA), and then resuspended in 33% Percoll (GE Healthcare, North Richland Hills, TX, USA). The suspension was centrifuged at 900×*g* for 30 min, and red blood cells (RBCs) were removed by RBC Lysing Buffer (Sigma-Aldrich, USA).

### Flow Cytometry

Single-cell suspension of HMNCs were isolated and freshly labeled with fluorochrome-conjugated antibodies, including antimouse CD45, CD11b, Gr-1, Ly6G, Ly6C, Ki67 (BD Bioscience), CD3, CD4, CD8, CD25, CD69, NK1.1, TCRβ (BioLegend, San Diego, CA, USA), and anti-IRF-8 (eBioscience, San Diego, CA, USA) antibodies. Antihuman LXR Alpha antibody used in flow cytometry was purchased from LSBio (LS-C223499, Seattle, WA, USA), and the rabbit IgG isotype control was from Novus (NBP2-36463APC, St. Louis, MO, USA). Cellular apoptosis was detected with FITC Annexin V Apoptosis Detection Kit (BD Bioscience). Intracellular staining was performed using the Fixation/Permeabilization Kit (BD Bioscience) and Transcription Factor Buffer Set (BioLegend) according to the manufacturer’s instructions. Flow cytometry was performed with LSR Fortessa (BD Bioscience), and data were analyzed using the FlowJo software version 10.0.2 (Three Star, San Carlos, CA, USA).

### MDSC Isolation and T-Cell Suppression Assay

MDSCs were magnetic sorted from the liver of LXRα^−/−^ or WT mice following 16 h ConA injection with MDSC Isolation Kit (Miltenyi Biotec, Auburn, CA, USA). T cells were obtained from the spleen of WT mice using Pan T Cell Isolation Kit (Miltenyi Biotec, USA). T cells labeled with CFSE (Invitrogen, Waltham, MA, USA) were activated with anti-CD3/CD28 beads (Miltenyi Biotec, USA) and further cocultured with purified liver MDSCs. The proliferation of T cells was assessed after 72 h and then analyzed with Flowjo software.

### Adoptive Cell Transfer

MDSCs were purified from the bone marrow of LXRα^−/−^ mice and WT mice treated with ConA for 3 h. Subsequently, 5 × 10^6^ MDSCs/mouse were transferred through tail-vein injection, and recipient WT mice were treated with ConA 1 h later. Mice were killed 16 h following ConA challenge and assessed for liver histology and transaminase levels.

### Generation of Bone Marrow-Derived MDSCs

Bone marrow cells were cultured with recombinant murine GM-CSF (40 ng/ml, PeproTech, Rocky Hill, NJ, USA) and IL-6 (40 ng/ml, PeproTech) in RPMI 1640 supplemented with 10% heat-inactivated FBS, 10 mM HEPES, 1 mM penicillin-streptomycin, and 50 mM 2-mercaptoethanol for 4 days. For LXR activation, 1 μM GW3965 was added at days 0 and 3.

### Transcriptional Sequencing

MDSCs were purified from bone marrow of WT mice (*n* = 3) and LXRα^−/−^ mice (*n* = 3) following 24 h injection of ConA with MDSC Isolation Kit (Miltenyi Biotec, USA). Total RNA was extracted from MDSCs using Trizol (Invitrogen). Transcriptome libraries were generated with the TruSeq RNA sample preparation kit (Illumina, San Diego, CA, USA), and sequencing was performed using the Illumina HiSeq X Ten instrument by the commercial service of Genergy Biotechnology Co. Ltd. (Shanghai, China).

### Quantitative Real-Time PCR

Total RNA was extracted with TRIzol Reagent (Invitrogen, USA) and cDNA was synthesized with PrimeScript™ RT Reagent Kit (Takara, Japan). Real-time PCR was performed using TB Green^®^ Fast qPCR Mix (Takara, Japan) on a StepOnePlus™ (Applied Biosystems, Waltham, MA, USA). Gene expression was normalized to the level of β-actin mRNA. The sequences for the primers used are as follows: murine IRF-8, forward-5′-GATCGAACAGATCGACAGCA-3′, reverse-5′-GCTGGTTCAGCTTTGTCTCC-3′; and β-actin, forward -5′-CTAAGGCCAACCGTGAAAAG-3′, reverse-5′-GGTACGACCAGAGGCATACA-3′.

### Western Blot

Primary antibodies applied in Western blot assay mainly include antibody against caspase-3 (#9662, Cell Signaling, Danvers, MA, USA), caspase-8 (#4790, Cell Signaling), PCNA (#13110, Cell Signaling), IRF-8 (#5628, Cell Signaling), and β-actin (#4970, Cell Signaling).

### Dual Luciferase Reporter Assay

Briefly, 1 × 10^4^ HEK293T cells were transfected with 10 ng Renilla luciferase plasmid, 100 ng firefly luciferase plasmid pGL3-IRF-8, and 100 g pCAGPuroAS05-NR1H3 plasmid using FuGENE^®^ HD Transfection Reagent (Promega, Madison, WI, USA). After 48 h, cell luciferase was measured by Dual-Glo^®^ Luciferase Assay System (Promega). Firefly luciferase activity was normalized to Renilla luciferase.

### Statistical Analyses

All analyses were performed using GraphPad Prism 6.0 software. Data were presented as the mean ± standard error (SEM). Statistical differences were determined by unpaired two-tailed *t*-tests, and significance was defined as *p > 0.05, **p < 0.01, ***p < 0.001, and ****p < 0.0001.

## Results

### LXRα Expression Was Elevated in AIH Patients and Colocalized With MDSCs

**Figure 1:**
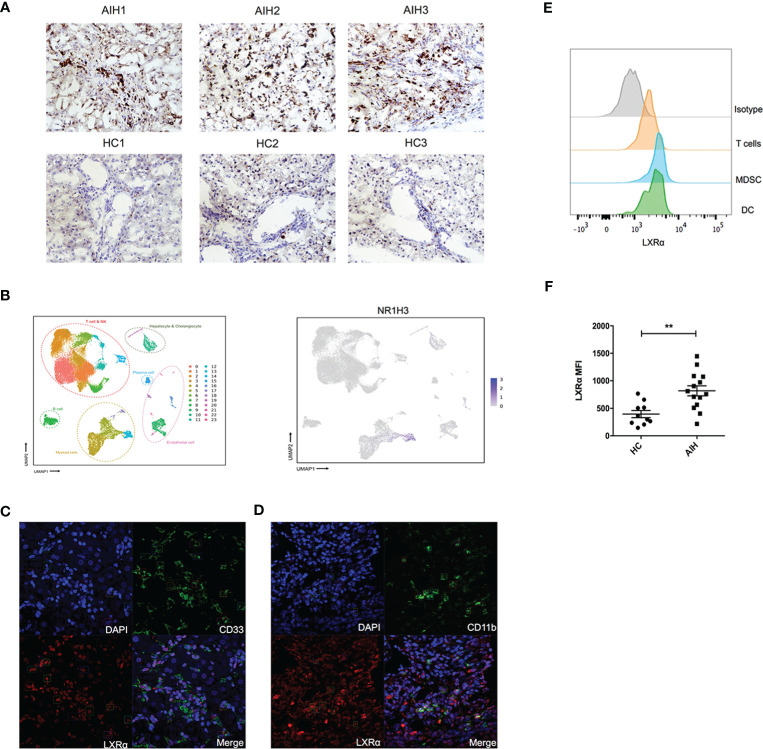
Expression of LXRα was elevated in the liver of AIH and colocalized with MDSCs. **(A)** Representative immunohistochemistry staining of LXRα (×100) in the frozen liver sections of patients with AIH and healthy controls. **(B)** Single-cell sequencing data (GSE136103) of hepatic nonparenchymal cells showed that LXRα is highly expressed by myeloid cells. Confocal microscopy demonstrated the colocalization of the MDSC markers CD33 **(C)** and CD11b **(D)** with LXRα using liver tissue of AIH patients. **(E)** Expression of LXRα in the peripheral CD3^+^ T lymphocytes, HLA-DR^−/lo^CD11b^+^CD33^+^ MDSCs, and CD11c^+^HLA-DR^+^ DCs of AIH patients and healthy controls was detected by flow cytometry. **(F)** Mean fluorescence intensity (MFI) of LXRα in peripheral blood mononuclear cells of AIH patients (*n* = 14) and healthy donors (*n* = 10) was calculated by MFI of LXRα minus MFI of isotype antibody. The AIH patients who provided peripheral blood were newly diagnosed and have not received treatment of steroid. Data are presented as mean ± SEM. **p < 0.01.

Human LXRα is known to upregulate its own expression upon activation. We explored the expression of this nuclear factor in liver tissue from AIH patients and healthy donors. Immunochemistry staining of the frozen sections revealed that expression of LXRα was substantially increased in AIH patients, compared with healthy controls (**Figure 1A**). We examined previously published single-cell RNA sequencing data of liver nonparenchymal cells (GSE136103) and found that LXRα tended to be highly expressed in the “myeloid cell” cluster (**Figure 1B**). Subsequently, we utilized confocal microscopy to investigate localization of LXRα and the surface markers of MDSCs in AIH patients, including CD11b and CD33. Indeed, LXRα was colocalized with CD11b and CD33 (**Figures 1C, D**). To further validate the cell source of LXRα, expression of LXRα in the peripheral blood from AIH patients and healthy donors was examined by flow cytometry. Consistently, LXRα was preferentially expressed in myeloid cells including MDSCs (HLA-DR^−/lo^CD11b^+^CD33^+^) (**Figure 1E**). Moreover, a higher expression of LXRα was observed in the circulating immune cells of AIH patients than healthy controls (**Figure 1F**).

### Deletion of LXRα Facilitated Expansion of Liver MDSCs and Ameliorated Hepatitis

**Figure 2:**
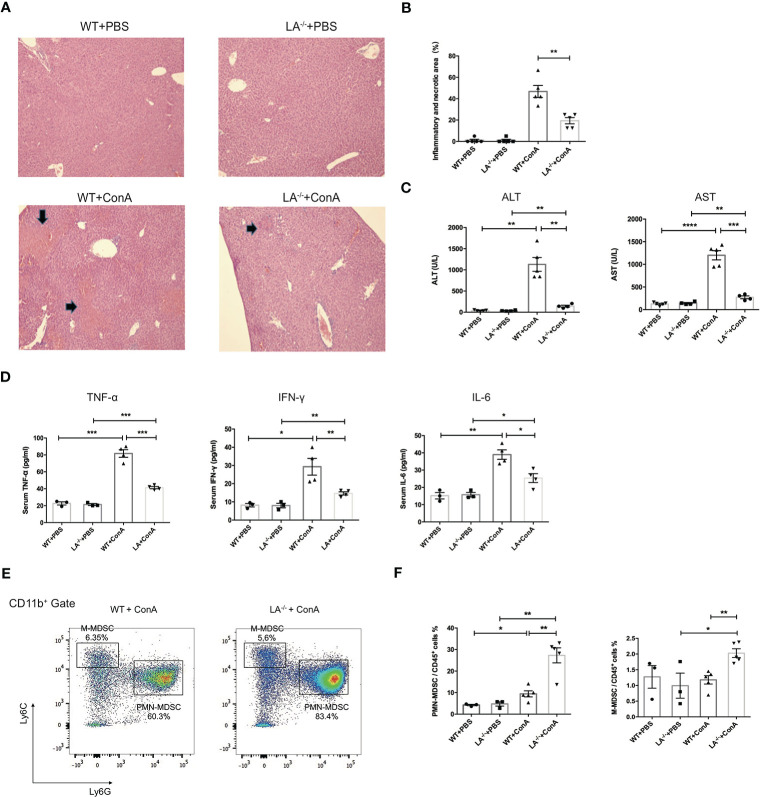
Knockout of LXRα mitigated ConA-induced hepatitis by facilitating expansion of MDSCs. **(A)** Representative H&E staining (×100) of livers in WT or LXRα^−/−^ mice injected i.v. with PBS or ConA. Mice were killed 24 h after ConA administration. **(B)** Quantification of inflammatory and necrotic area in each group. **(C)** Serum levels of ALT and AST. **(D)** Serum levels of inflammatory cytokines, including TNF-α, IFN-γ, and IL-6. **(E)** Liver MNCs harvested from WT and LXRα^−/−^ mice at 18 h after ConA challenge were analyzed by flow cytometry for the frequency of MDSCs. **(F)** Quantification of hepatic PMN-MDSCs and M-MDSCs in each group (*n* = 3–6 per group). Data are presented as mean ± SEM. *p > 0.05, **p < 0.01, ***p < 0.001, and ****p < 0.0001.

To investigate the potential role of LXRα in MDSCs *in vivo*, wild-type (WT) and LXRα^−/−^ knockout (LXRα^−/−^) mice were challenged with ConA, respectively. ConA-induced hepatitis has been widely used as murine model of AIH and elicits rapid recruitment of MDSCs to the liver (7). Interestingly, hepatic area of inflammation and necrosis were significantly attenuated in mice deficiency of LXRα following ConA injection (**Figures 2A, B**), along with decreased levels of ALT and AST (**Figure 2C**). Levels of peripheral inflammatory cytokines, including IFN-γ, TNF-α, and IL-6, increased after ConA induction but were markedly lower in LXRα^−/−^ mice than WT controls (**Figure 2D**), confirming an ameliorated immune response upon LXRα deletion. Notably, PMN-MDSCs (CD11b^+^Ly6G^+^Ly6C^lo^ cells) and M-MDSCs (CD11b^+^Ly6G^−^Ly6C^high^ cells) were substantially expanded in the liver of LXRα^−/−^ mice, compared with WT mice (27.3% *vs*. 9.42%, *p* < 0.01; 2.02% *vs*. 1.18%, *p* < 0.01; **Figures 2E, F** **)**. MDSCs were reported to have potent immunosuppressive capacity, which probably explain the mitigated inflammation and less tissue injury upon LXRα ablation. In line, a decreased activation of T lymphocytes was observed in LXRα^−/−^ mice (**Supplementary Figures S1A, B**). There was no significant difference with regard to the frequency of T-regulatory (Treg) cell or macrophage between WT and LXRα^−/−^ mice (**Supplementary Figures S1C, D**). Subsequent antagonizing LXRα with SR9243 also resulted in an increased accumulation of hepatic MDSCs and simultaneously ameliorated liver injury (**Supplementary Figure S2**).

### LXRα Ablation Enhanced the Proliferation and Survival of MDSCs in Inflamed Liver

**Figure 3:**
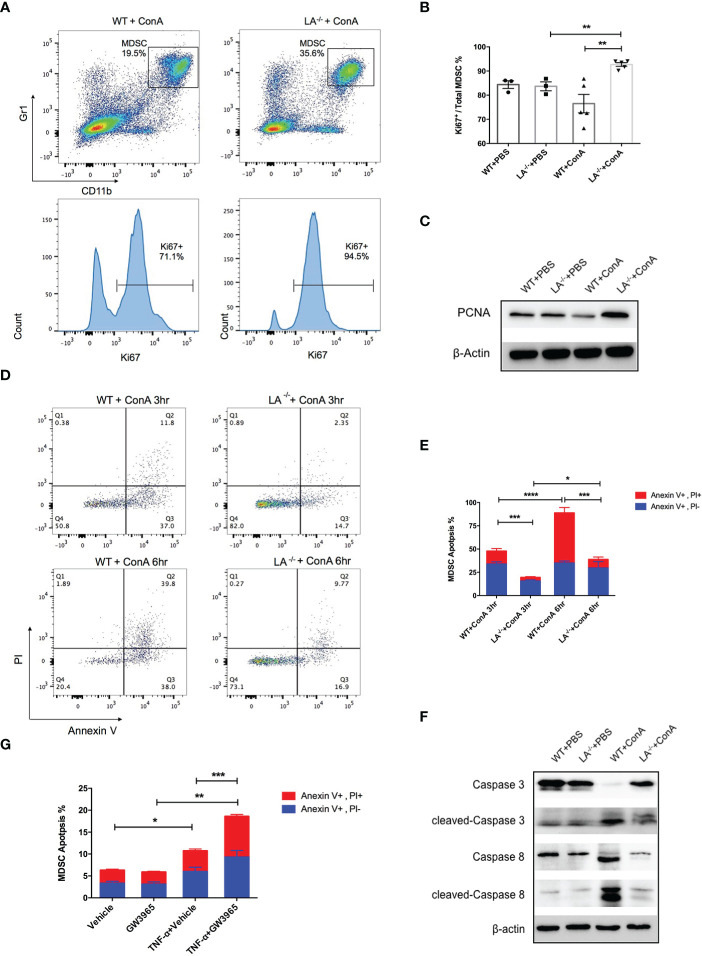
Deletion of LXRα-favored proliferation and survival of MDSCs. **(A)** Representative flow cytometric images of ki67 expression in hepatic MDSCs in WT and LXRα^−/−^ mice following an 18-h ConA treatment. **(B)** Statistic analyze of Ki67^+^ percentage in MDSCs in each group. **(C)** Expression of PCNA in MDSCs was measured by Western blot. **(D, E)** Hepatic MDSCs were gated and further analyzed for the staining of Annexin V and PI at the time of 3 and 6 h after ConA injection. Annexin V^+^, PI^−^ cells were early apoptotic, while Annexin V^+^, PI^+^ cells were regarded as late apoptotic. **(F)** MDSCs purified from WT and LXRα ^−/−^ mice were bulked and analyzed for the activation of caspase-3 and caspase-8 pathways by Western blot. **(G)** MDSCs isolated from WT and LXRα^−/−^ mice were *in vitro* treated with or without 10 μM LXR agonist GW3965 for 24 h, and 20 ng/ml TNF-α was further added to induce cell apoptosis. All data are presented as mean ± SEM. *p > 0.05, **p < 0.01, ***p < 0.001, and ****p < 0.0001.

To further explore the mechanisms of MDSC accumulation in LXRα^−/−^ mice, we examined the effects of LXRα knockout on proliferation and apoptosis of MDSCs. Intriguingly, CD11b^+^Gr-1^+^ MDSCs in the liver of LXRα^−/−^ ConA group possessed significantly higher frequency of ki67-positive cells than WT controls (92.7% *vs*. 76.5%, *p* < 0.01, **Figures 3A, B**), which was supported by elevated expression of PCNA in MDSCs of LXRα^−/−^ mice treated with ConA (**Figure 3C**). It is known that peripheral MDSCs are prone to programmed cell death. In concordance, MDSCs in the liver of WT mice group exhibited substantial apoptosis as early as 3 h following ConA challenge, which further upregulated at the time point of 6 h. Conversely, MDSCs in LXRα^−/−^ ConA group showed much lower frequency of cell apoptosis, both at 3 and 6 h (19.45% *vs*. 47.81%, *p* < 0.001; 38.82% *vs*. 88.75%, *p* < 0.001, **Figures 3D, E**). Western blot assay confirmed an excessive activation of apoptosis signaling pathway in the MDSCs of WT group, as evidenced by the cleavage of caspase-8 and caspase-3 (**Figure 3F**). In parallel, MDSCs treated with LXR agonist (GW3965) *in vitro* were more susceptible to cell death induced by TNF-α (*p* < 0.01, **Figure 3G**).

### MDSCs Protected Liver From Immune-Mediated Injury

**Figure 4:**
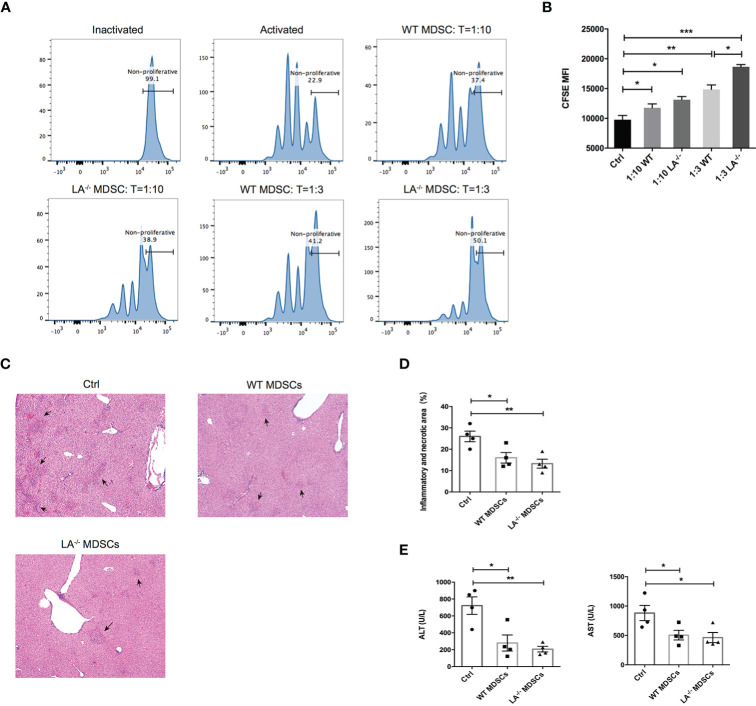
MDSCs expanded in murine model of ConA possess immunosuppressive function and prevented T-cell-mediated liver injury. **(A, B)** CFSE of proliferating T cells cocultured with MDSCs at different ratios (10:1 and 3:1). MDSCs were purified and bulked from liver of WT and LXRα^−/−^ mice after 16-h treatment of ConA. To satisfy the cellular amount for adoptive transfer experiment, MDSCs were magnetic sorted from the bone marrow of WT and LXRα^−/−^ mice challenged with ConA for 3 (h) Each recipient WT mice was injected 5 × 10^6^ MDSCs and 1 h later treated with ConA. Mice that received PBS were used as positive control. **(C, D)** Liver histology and the quantification of inflammatory and necrotic area. **(E)** Serum levels of ALT and AST. Data are presented as mean ± SEM. *p > 0.05, **p < 0.01 and ***p < 0.001.

To confirm the immunosuppressive effects of MDSCs in ConA-induced hepatitis, we isolated the hepatic MDSCs from LXRα^−/−^ and WT mice respectively and cocultured the MDSCs with T cells activated by anti-CD3/CD28 beads at different effector-and-target ratios. As expected, MDSCs purified from both LXRα^−/−^ and WT groups effectively suppressed the proliferation of T cells at both 1:3 and 1:10 ratios (MDSC:T cell) (**Figures 4A, B**). More importantly, ConA-induced MDSCs in LXRα^−/−^ mice exhibited slightly higher immunosuppressive capacity than that of WT controls (**Figure 4B**). In the next adoptive transfer experiment, mice were protected from ConA-mediated hepatitis by prior transfer of MDSCs from both WT and LXRα^−/−^ mice (**Figures 4C–E**). All the above findings support that MDSCs exert potent immunoregulatory role under LXRα knockout background and thereby efficiently protect liver from immune-mediated tissue injury.

### LXRα Regulated MDSCs Negatively Through Transcription Activation of IRF-8

**Figure 5:**
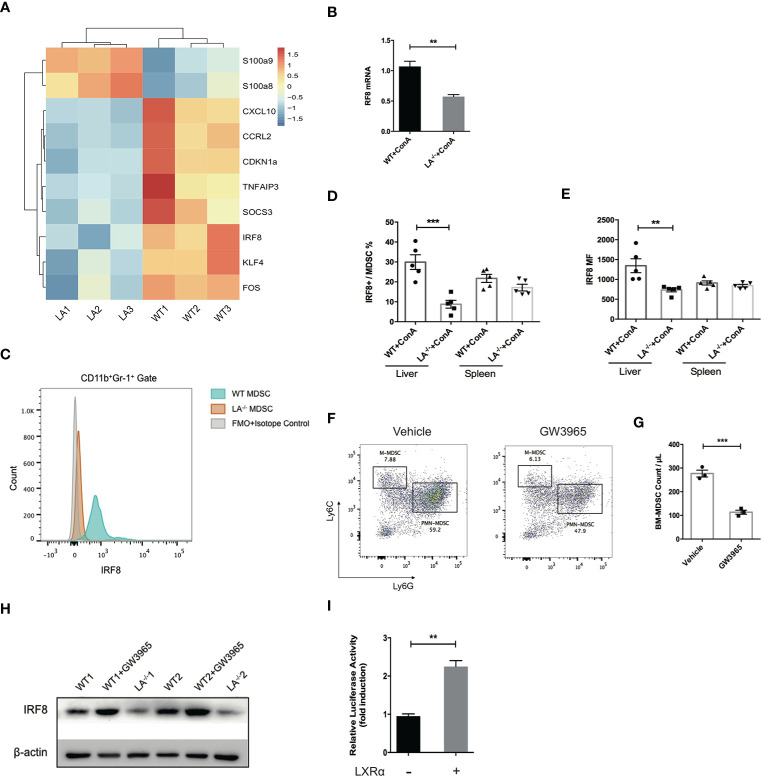
LXRα activation induced IRF-8 expression and hampered the differentiation of MDSCs. **(A)** MDSCs purified from bone marrow of WT and LXRα^−/−^ mice treated with ConA (*n* = 3 per group) were subjected for transcriptional sequencing. Shown are the representative differential genes. IRF-8 was downregulated in MDSCs of LXRα^−/−^ mice. **(B)** Validation of IRF-8 expression in MDSCs from WT and LXRα^−/−^ mice by real-time PCR. **(C–E)** Protein expressions of IRF-8 in hepatic MDSCs were confirmed by flow cytometry. **(F, G)** Induction of MDSCs from bone marrow cells using GM-CSF and IL-6 for 4 days. BM-derived MDSCs were reduced significantly by treatment of GW3965. **(H)** BM-derived MDSCs from WT and LXRα^−/−^ mice, and WT mice treated with GW3965 were analyzed for IRF-8 expression by Western blot. **(I)** The IRF-8 promoter luciferase activity with or without overexpression of LXRα after 48-h transfection in HEK293T cells. The experiment has been repeated for three times. Data are presented as mean ± SEM. **p < 0.01, ***p < 0.001.

IRF-8 has been well characterized as a key factor during the differentiation and maturation of myeloid cells. Mice defect in IRF-8 generate massive amount of MDSCs, while overexpression of IRF-8 led to depletion of MDSCs in murine models of carcinoma, indicating IRF-8 as a negative regulator in MDSC biology (3, 23, 24). By transcriptome sequencing of MDSCs isolated following ConA treatment, we noticed that the expression of IRF-8 in LXRα^−/−^ mice was significantly lower than its WT counterparts (**Figure 5A**). Additionally, S100A8 and S100A9, transcription factors known to induce MDSC differentiation, were upregulated in LXRα^−/−^ group. Lower expression of IRF-8 mRNA in the MDSCs from LXRα^−/−^ mice was validated by quantitative PCR (**Figure 5B**). Flow cytometry confirmed that hepatic MDSCs in LXRα^−/±^ mice exhibited much lower level of IRF-8 than WT mice challenged with ConA. However, such difference was not observed in spleen MDSCs (**Figures 5C–E**).

We next use cytokines to induce MDSCs from bone marrow cells. LXR agonist resulted in an impairment of MDSC generation, particularly PMN-MDSCs (**Figures 5F, G**). As expected, expression of IRF-8 decreased markedly over the 4-day induction by GM-CSF and IL-6. Consistent with the *in vivo* data, lower expression of IRF-8 was detected in bone marrow (BM)-derived MDSCs from LXRα^−/−^ mice than WT counterpart, whereas WT MDSCs treated with LXR agonist showed upregulation of IRF-8 expression (**Figure 5H**). Next, dual-luciferase reporter assay was conducted to identify functional interactions between LXRα and the promoter of IRF-8. Overexpression of LXRα led to twofold increase of the luciferase activity in HEK293T cells 48 h after transfection (**Figure 5I**), further supporting the transcriptional regulation of IRF-8 by LXRα.

## Discussion

In the current study, we investigated the impacts of LXRα on the differentiation and function of MDSCs in inflammatory liver milieu. By utilizing the model of ConA-induced hepatitis, we showed that increased MDSCs were generated in LXRα^−/−^ mice and exerted immunosuppressive effects to ameliorate liver inflammation. Given that LXRα was highly expressed in AIH patients, inhibition of the nuclear factor selectively represented a novel strategy for immune treatment of AIH.

Emerging studies have characterized the implication of LXR in hepatic inflammation and innate and adaptive immunity (11). Nonetheless, the anti-inflammatory or proinflammatory role of LXR still remains controversial. Function of these NRs are various depending on the different cell types and disease context (25). With regard to myeloid differentiation, it has been shown that overexpression of LXRα promoted maturation of DCs and endowed it with enhanced ability to stimulate T-cell proliferation (26). Conversely, a recent study reported that 27-hydroxycholesterol (27HC), one of the oxysterols enriched in tumor site, impaired T-cell proliferation and cytotoxicity by acting on myeloid cells in a LXR-dependent manner (27). For immature myeloid cells, our data are in accordance with a recent tumor study showing that LXR agonism boosted T-cell-mediated anticancer immunity by specifically depleting MDSCs (28). The LXR agonist has undergone phase I study and the mechanism work consistently in patients with solid tumors (28).

IRF-8 is an integral transcriptional factor during myeloid differentiation and lineage commitment (24). It has been demonstrated that deletion of IRF-8 led to uncontrolled expansion of MDSCs (23). Furthermore, low expression of IRF-8 conferred peripheral MDSCs with increased resistance to apoptosis (29). In our experiment, knockout of LXRα resulted in increased accumulation of MDSCs in response to the acute hepatitis, and it appeared that activation of LXR in MDSCs promoted its apoptosis. Combining with the transcriptome data, we focused on the possible interactions of LXRα and IRF-8 in MDSCs. Indeed, liver MDSCs (CD11b^+^Gr1^+^) in LXRα^−/−^ mice manifested significant lower expression of IRF-8. Treatment of LXR agonist upregulated the expression of IRF-8 in primary MDSCs *in vitro*. Furthermore, direct binding of LXRα to the promoter region of IRF-8 has been validated by Chip assay in mouse monocyte cell line in a previous study (30). Therefore, we concluded that ablation of LXRα promoted generation of MDSCs *via* downregulating IRF-8.

Unlike ubiquitously expressed LXRβ, LXRα is selectively expressed in metabolically active tissue and cell types. Despite the high homology of the sequence, recent studies have identified differential genes targeted by LXRα and LXRβ (31). LXRα, for example, preferentially regulates genes concerning leukocyte apoptosis and migration, whereas LXRβ is more related with differentiation of lymphocytes (31, 32). The hepatotoxicity of ConA has been mainly attributed to activation of T lymphocytes (2). In fact, we found that LXRα but not LXRβ knockout mice were resistant to the phenotype of hepatitis (**Supplementary Figure S3**). LXR activation, in particular LXRβ, has been shown to suppress Th1 and Th17 polarization and skewed the differentiation of Treg cells (33, 34). Moreover, defect in LXR is known to promote the proliferation of T lymphocytes (35). In our experiment, however, an impaired activation of T lymphocytes was observed in LXRα^−/−^ mice treated with ConA. The contradictions above, to an extent, excluded the direct effects of LXRα on lymphocytes in the model.

It has been reported that activation of human LXRα upregulates its own transcription (36). Herein elevated expression of LXRα observed in AIH may be attributed to the abnormal activation of the nuclear receptor. Oxysterols, the endogenous ligands of LXR, are cholesterol metabolites produced by enzymatic reactions or oxidation *via* reactive oxygen species (ROS) (37). Perturbations of oxysterol has been described in various autoimmune and inflammatory diseases, including multiple sclerosis, inflammatory bowel disease, rheumatic arthritis, and nonalcoholic fatty liver disease (37–41). Mechanistically, the expression levels of the hydroxylases, enzymes responsible for production of oxysterol, can be upregulated by inflammatory signals. Accordingly, LPS and interferons promoted the synthesis and release of 25-hydroxycholesterol by macrophage and DC, which then further amplified the inflammatory reactions (42, 43). Another important connection may lie in the fact that AIH is associated with increased oxidative stress in liver (44), where some oxysterol species can be nonenzymatically synthesized *via* ROS. Nevertheless, the oxysterol metabolism in AIH and its relationship with disease progression needs to be further investigated.

In line with our data, a recent study reported that consecutive activation of LXRα exacerbated ConA-induced hepatitis (45), which supported a pathogenic role of LXRα during AIH development. Additionally, LXRα^−/−^ mice fed with a high-fat and high-cholesterol diet were resistant to ConA due to the dysfunction of invariant NKT cells (46). Our previous work has emphasized the therapeutic potential of MDSCs in autoimmune liver diseases (8, 9). Increased frequencies of MDSCs were observed in patients of AIH and PBC, which was supposed to be a negative feedback to liver inflammation. Herein, by adoptive transferring MDSCs purified from WT or LXRα^−/−^ mice, we showed that MDSCs generated in response to hepatitis were sufficient to protect against the T-cell-mediated liver injury. In this regard, it seems plausible to antagonize LXRα for countering the excessive immune responses in AIH.

In conclusion, LXRα was highly expressed in the myeloid cells of AIH. LXRα deficiency facilitated the expansion of MDSCs in response to immune-mediated hepatitis and therefore alleviated liver injury. Activation of LXR, in contrast, impaired the differentiation of MDSCs and rendered MDSCs more prone to apoptosis, probably by transcriptional regulation of IRF-8. Considering the potent immunosuppressive capacity of MDSCs, our study provided rationales to pharmacologically modulate LXRα activity for treating AIH.

## Data Availability Statement

The original contributions presented in the study are included in the article/**Supplementary Material**. Further inquiries can be directed to the corresponding author.

## Ethics Statement

The studies involving human participants were reviewed and approved by the Ethics Committee of Renji Hospital. The patients/participants provided their written informed consent to participate in this study. The animal study was reviewed and approved by the Institutional Animal Care and Use Committee of Shanghai Jiao Tong University.

## Author Contributions

XM and RT conceptualized and supervised the study. XM, RT, and ML acquired the funding. XM and RT managed the resources. ML, BL, and JZ developed the methodology. BL, YL, QQ, and QL performed the investigation. BL and ML wrote the manuscript. RT and XM reviewed and edited the manuscript. All authors contributed to the article and approved the submitted version.

## Funding

This work was supported by the National Natural Science Foundation of China grants (#81830016, 81771732, and 81620108002 to XM; #81922010, 81873561, and 81570469 to RT; #81800504 to ML), Shanghai Sailing Program (No. 18YF1412900 to ML), and “Chen Guang” project supported by Shanghai Municipal Education Commission and Shanghai Education Development Foundation (No. 19CG16).

## Conflict of Interest

The authors declare that the research was conducted in the absence of any commercial or financial relationships that could be construed as a potential conflict of interest.

## Publisher’s Note

All claims expressed in this article are solely those of the authors and do not necessarily represent those of their affiliated organizations, or those of the publisher, the editors and the reviewers. Any product that may be evaluated in this article, or claim that may be made by its manufacturer, is not guaranteed or endorsed by the publisher.

## Abbreviations

AIH, autoimmune hepatitis; AILD, autoimmune liver diseases; AP-1, activator protein-1; ALT, alanine aminotransferase; AST, aspartate aminotransferase; ConA, concanavalin A; CCR7, C-C chemokine receptor type 7; DC, dendritic cell; GM-CSF, granulocyte-macrophage colony-stimulating factor; HLA, human leukocyte antigen; IRF-8, interferon regulatory factor 8; IFN-γ, elevation of interferon-γ; IL-6, interleukin-6; LXR, liver X receptor; LPS, lipopolysaccharide; MDSC, myeloid-derived suppressor cell; NR, nuclear receptor; NF-κB, nuclear factor κ-light-chain-enhancer of activated B cells; PCNA, proliferation cell nuclear antigen; SPF, specific pathogen free; TNF-α, tumor necrosis factor-α.
