# Supplementary material for: Myeloid-Derived Suppressive Cells Deficient in Liver X Receptor α Protected From Autoimmune Hepatitis
Source: Front Immunol. 2021 Aug 26;12:732102. doi: 10.3389/fimmu.2021.732102 (PMC8427166; doi:10.3389/fimmu.2021.732102)
Supplement: Supplementary file 1 [file DataSheet_1.pdf]

Supplementary Table 1. Clinical characteristics of AIH and healthy controls who provided peripheral blood samples for flow cytometry tests.

	AIH (n=14)	HC (n=10)
Age (years)	46.9±2.7	42.3±2.0
Gender (F/M)	13/1	8/2
ALT (U/L)	101.7±28.5	NA
AST (U/L)	149.9±30.4	NA
AKP(U/L)	137.4±20.7	NA
GGT (U/L)	103.8±24.1	NA
TBIL (μmol/L)	34.7±16.1	NA
IgG (g/L)	20.99±1.7	NA

F/M, female and male; NA, not applicable; data were shown as mean ± standard error (SEM).

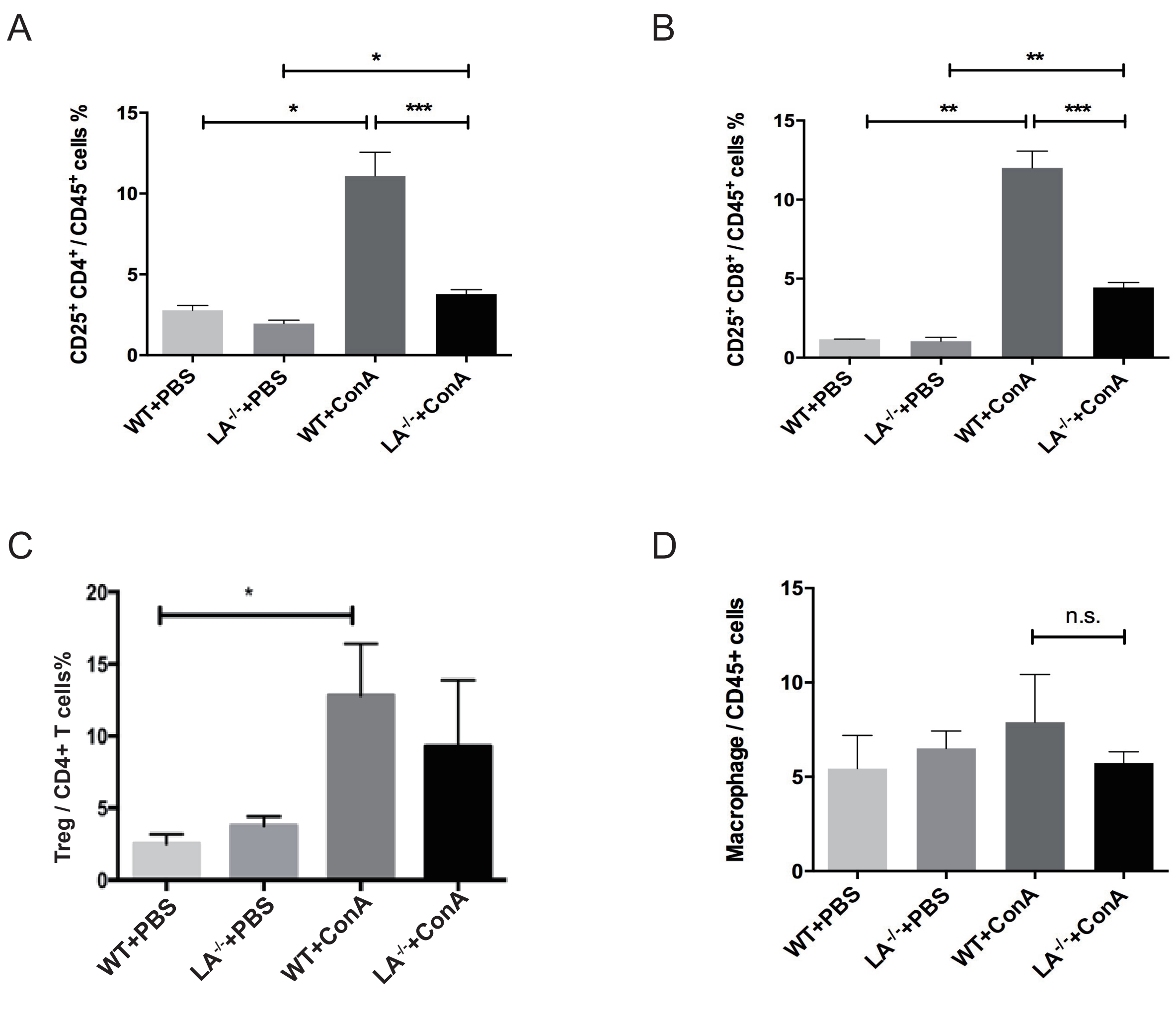

Supplementary Figure 1.Flow cytometry analysis of liver mononuclear cells after 24-hour treatment of ConA. A decresaed frequency of liver (A) $CD25^{+}CD4^{+}$ T cells and (B) $CD25^{+}CD8^{+}$ T cells was observed in LXRα$^{-/-}$ mice. However, there is no significant difference in liver (C) Tregs or (D) macrophages bewteen WT and LXRα$^{-/-}$ mice.

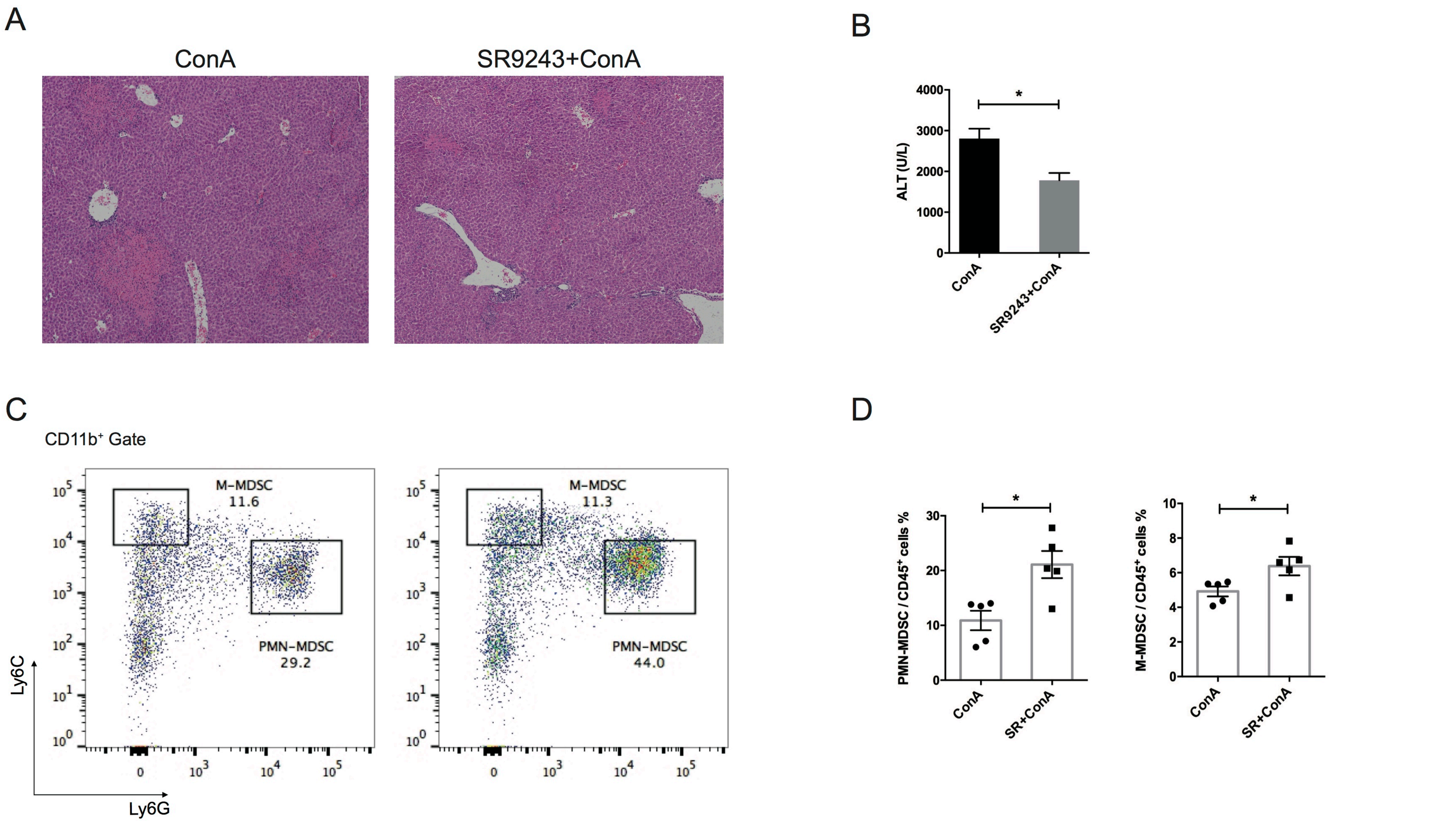

Supplementary Figure 2. Inhibition of LXR promoted expansion of hepatic MDSCs. To antagonizing LXR, WT mice were given LXR inverse agonist SR9243 (30mg/kg, Selleckchem) i.p. twice at 24h and 1h before ConA treatment. (A) Representative H&E staining (×100) of livers WT mice treated with vehicle or SR9243. (B) Serum levels of ALT. (C, D) Hepatic PMN-MDSCs and M-MDSCs were investigated by flow cytometry.

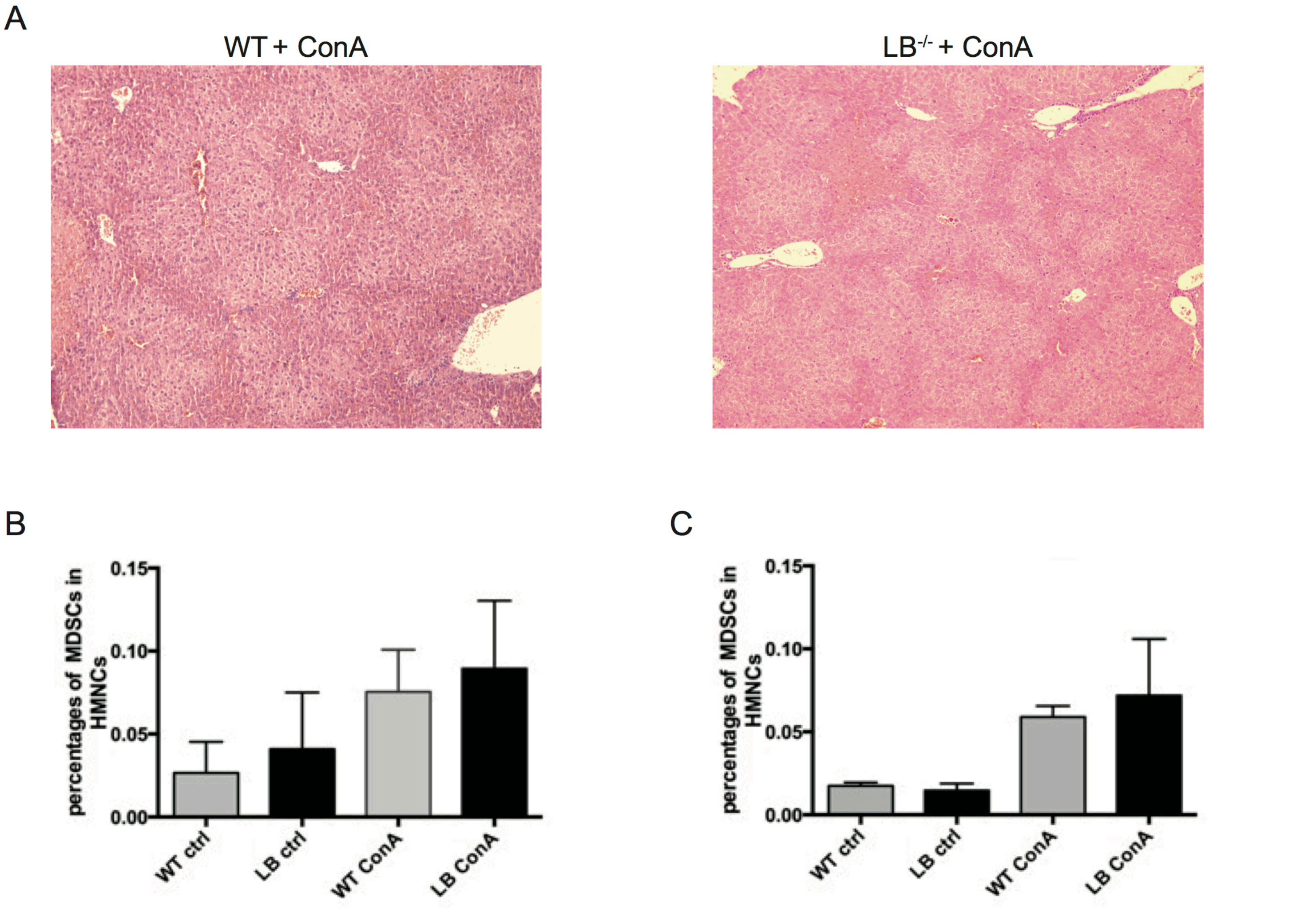

Supplementary Figure 3. Deficiency in LXRβ exhibited no impacts on ConA-induced hepatitis or expansion of MDSCs. (A) Representative H&E staining (×100) of livers in WT or LXRβ -/- mice injected i.v. with ConA for 24h. Percentage of liver (B) PMN-MDSC and (C) M-MDSC in WT and LXRβ knockout mice following treatment of PBS or ConA.
